# Coordinated Induction of Antimicrobial Response Factors in Systemic Lupus Erythematosus

**DOI:** 10.3389/fimmu.2019.00658

**Published:** 2019-04-04

**Authors:** Prathapan Ayyappan, Robert Z. Harms, Jane H. Buckner, Nora E. Sarvetnick

**Affiliations:** ^1^Department of Surgery-Transplant, University of Nebraska Medical Center, Omaha, NE, United States; ^2^Translational Research Program, Benaroya Research Institute, Seattle, WA, United States; ^3^Mary and Dick Holland Regenerative Medicine Program, University of Nebraska Medical Center, Omaha, NE, United States

**Keywords:** antimicrobial proteins, SLE, EndoCAbs, sCD14, lysozyme, lipopolysaccharide binding protein, fatty acid binding protein, CXCL16

## Abstract

Systemic lupus erythematosus (SLE) is a chronic autoimmune disease characterized by dysregulated autoantibody production and complement activation leading to multi-organ damage. The disease is associated with increased intestinal permeability. In this study, we tested the hypothesis that SLE subjects have increased systemic exposure to bacteria. Since bacteria induce the expression of antimicrobial response factors (ARFs), we measured the levels of a series of clinically relevant ARFs in the plasma of SLE subjects. We found that levels of sCD14, lysozyme, and CXCL16 were significantly elevated in SLE subjects. A strong positive correlation was also observed between sCD14 and SELENA-SLEDAI score. Interestingly, the ratio of EndoCAb IgM:total IgM was significantly decreased in SLE and this ratio was negatively correlated with sCD14 levels. Although, there were no significant differences in the levels of lipopolysaccharide binding protein (LBP) and fatty acid binding protein 2 (FABP2), we observed significant positive correlations between lysozyme levels and sCD14, LBP, and FABP2. Moreover, galectin-3 levels also positively correlate with lysozyme, sCD14, and LBP. Since our SLE cohort comprised 43.33% males, we were able to identify gender-specific changes in the levels of ARFs. Overall, these changes in the levels and relationships between ARFs link microbial exposure and SLE. Approaches to reduce microbial exposure or to improve barrier function may provide therapeutic strategies for SLE patients.

## Introduction

Systemic lupus erythematosus (SLE) is a heterogeneous chronic autoimmune disease, primarily affecting women with a gender bias of 9:1 (1). The initiating stimulus of SLE is unknown. SLE is characterized by dysregulated autoantibody production and complement activation. Target tissues include the central nervous system (CNS), kidneys, blood, skin, and joints (2). Diagnosis of SLE is based on clinical manifestations, mainly in the skin and musculoskeletal tissues. A large proportion of subjects present with either hyperkeratosis, maculopapular exanthema, synovitis, myalgia, or arthralgia (3). Due to its heterogeneous nature and diverse clinical manifestations, it is difficult to accurately diagnose SLE (4). Genetic susceptibility as well as environmental and epigenetic factors contribute to the pathogenesis of this disease (1, 5, 6).

Recent reports suggest that intestinal barrier defects and exposure to microbial products play an important role in the pathology of SLE (7–9). Furthermore, exposure to products from Gram-negative bacteria such as LPS aggravate SLE (10). It has been postulated that products from both Gram-negative and Gram-positive bacteria act as initiating or accelerating factors for this disease (11–13). A significant alteration in the gut microbiome was also observed in human SLE (7, 14, 15). Alterations in the intestinal microbiome composition and leakage into the body could promote a toxic inflammatory microenvironment, leading to loss of self-tolerance and autoimmunity (14–16).

Exposure to microbes and their products elicits the production of antimicrobial response factors (ARFs). ARFs comprise the first line of defense against infection. ARFs include proteases, cytokines, chemokines, and peptides (17). ARFs directly kill bacteria and/or activate innate immunity (18) by recruiting neutrophils and macrophages. This facilitates rapid microbial clearance, and ultimately reduces inflammation (19).

In the present study, we tested the hypothesis that SLE subjects have increased exposure to bacteria. We asked whether these subjects exhibited heightened circulating levels of ARFs. To query this, we measured the levels of a series of representative ARFs in plasma from female and male SLE subjects. The tested factors include sCD14, lipopolysaccharide-binding protein (LBP), EndoCAb IgG, IgM, and IgA, lysozyme, galectin-3, CXCL16, and LL-37. We also measured fatty acid binding protein-2 (FABP2) levels, since this reflects intestinal damage (20). Our results demonstrate a marked elevation of sCD14, lysozyme and CXCL16 in SLE subjects. In addition, we observed a reduction in the levels of EndoCAb IgM, suggesting acute bacterial exposure. We discovered significant correlations between lysozyme and sCD14 levels, and these factors also correlated with LBP, galectin-3 and FABP2, suggesting a common stimulus.

## Materials and Methods

### Study Subjects

Ethical approval for this study was obtained from Benaroya Research Institute's Institutional Review Board (#07109-136) in compliance with Declaration of Helsinki. Informed consents were obtained from each participant prior to including them in the study. Our study involved 30 SLE patients (13 males and 17 females), all fulfilling the revised classification criteria for SLE from the American College of Rheumatology (21) and 30 age and gender matched healthy control subjects (16 females and 14 males) with no personal or family history of autoimmunity. Exclusion criteria included history of recent infection and the use of steroids from the past 6 months. Out of 30 SLE subjects, 19 were treated with immunosuppressive and/or immunomodulatory drugs, which include mycophenolate mofetil, methotrexate, azathioprine, and hydroxychloroquine.

### Sample Collection

Venous blood was drawn from both SLE and control individuals in BD Vacutainer® K2 EDTA tubes (Franklin Lakes, NJ, USA). The collected whole blood was centrifuged (for 20 min at 3000 × g and 20°C) and then the plasma layer was removed. All the plasma samples were divided into multiple aliquots and were flash frozen in dry ice and stored at −80°C until analysis. Patient data is provided in Table 1. Individual ARF values, disease activity, drug use, and ANA titres are given in Supplementary Tables 1–3.

**Table 1:** Characteristics of SLE patients and healthy controls.

		**SLE Patients (*n* = 30)**	**Healthy controls (*n* = 30)**
	Mean age in years (range)	45.90 ± 13.04 (30–71)	46.13 ± 13.00 (26–68)
Sex (n)	Female	17	16
	Male	13	14
	Mean SELENA-SLEDAI^#^ score (range)	4.90 ± 5.98 (0–26)	–
	ANA^*^ titer (range)	1:80 to >1:640	–

*Values expressed as mean ± standard deviation (SD) wherever it is applicable*.

# *SELENA-SLEDAI- Safety of Estrogens in Lupus Erythematosus National Assessment-systemic lupus erythematosus disease activity index*.

* *ANA-antinuclear antibodies*.

### Measurement of Analytes in the Plasma

EndoCAb IgG, EndoCAb IgM, and EndoCAb IgA were measured using direct ELISA kits procured from Hycult Biotech. LBP and LL-37 were measured using sandwich ELISA kits procured from Hycult Biotech (Pennsylvania, USA). sCD14 and FABP2 were measured using sandwich ELISA kits from R&D systems (Minneapolis, USA). CXCL16 was analyzed using a sandwich ELISA kit procured from Thermo Scientific (Frederick, MD, USA). Galectin-3 was measured using sandwich ELISA kit (Abcam, MA, USA). Total IgG, IgA, and IgM were measured using sandwich ELISA kits procured from Invitrogen (Carlsbad, CA, USA). The samples were diluted in appropriate buffers, which contains HeteroBlock (Omega Biologicals, Bozeman, MT, USA) to block non-specific antibodies which may interfere with the assay. All the analyses were performed blinded to clinical status with the exception of galectin-3 and total immunoglobulins IgG, IgA, and IgM.

### Statistical Analysis

We transformed the data into logarithmic values for statistical analysis and correlation studies. For testing statistical significance, the unpaired *t*-test was used. For correlation analysis, Pearson product-moment correlation coefficient (Pearson's r) analysis was performed. For all statistical tests, *P* < 0.05 was considered to be statistically significant. All the statistical tests were done with GraphPad Prism 7 (GraphPad Software, Inc.).

## Results

### sCD14 Levels Are Increased in SLE Patients

**Figure 1:**
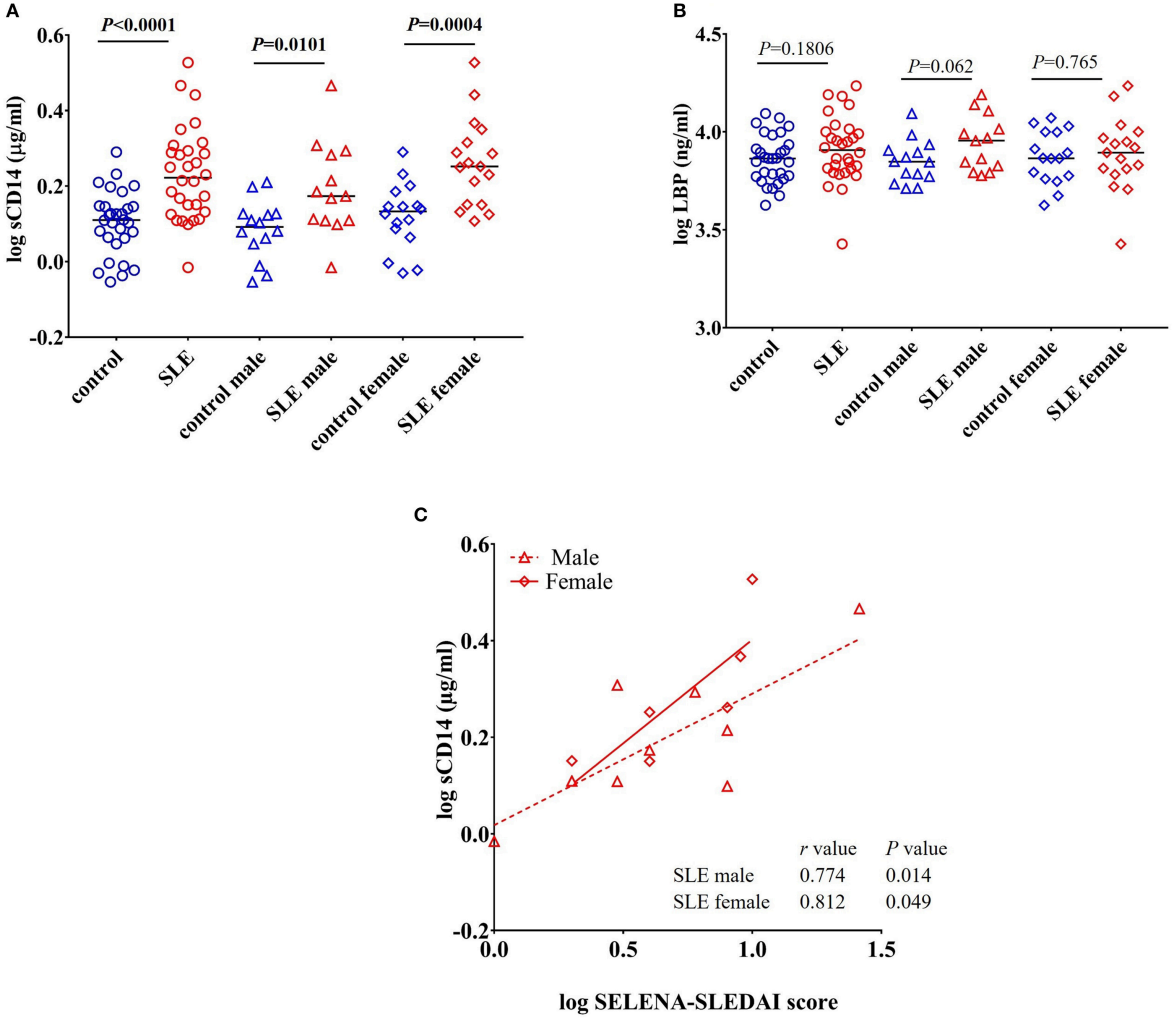
Increased levels of sCD14 in SLE subjects. **(A)** Concentrations of circulating sCD14 in healthy controls and SLE patients. **(B)** Concentrations of LBP in healthy controls and SLE patients. Bars represent median analyte levels. **(C)** Correlation between circulating sCD14 levels and the SELENA-SLEDAI score in SLE patients. Correlation studies showed a significant positive correlation between SLEDAI vs. sCD14 (*n* = 20).

CD14 is a co-receptor for LPS and is expressed by monocytes and macrophages (22). Upon stimulation with endotoxins, CD14 is shed from the cell surface leading to increased circulatory levels of soluble CD14 (sCD14). Increased levels of sCD14 could reflect endotoxin exposure and/or monocyte/macrophage activation (23). We found a significant increase in the levels of sCD14 (*P* < 0.0001) in SLE patients compared to control subjects (Figure 1A). We also compared sCD14 levels in male and female SLE subjects. Both SLE males and females showed significant elevations in sCD14 compared to controls (*P* = 0.0101 and *P* = 0.0004 for males and females, respectively). LBP binds to LPS and transfers LPS to CD14, leading to the activation of TLR4 (22, 24). We did not detect a significant difference in LBP levels in SLE patients compared to healthy controls (*P* = 0.180). However, SLE males showed a trend toward increased levels (*P* = 0.062), whereas SLE females were not different than controls (*P* = 0.765) (Figure 1B).

### sCD14 Positively Correlates With SELENA-SLEDAI

Safety of Estrogens in Lupus Erythematosus National Assessment-Systemic Lupus Erythematosus Disease Activity Index (SELENA-SLEDAI) is a modified version of the measure of disease activity, which is used as a clinical index for the severity of the disease (25, 26). SLEDAI levels reflect alterations in mucocutaneous, musculoskeletal, neurological, cardiopulmonary, renal, and hematological systems (27). Since we found an elevation in sCD14 in SLE patients, we asked whether sCD14 and SELENA-SLEDAI levels were correlated in SLE patients. Interestingly, sCD14 levels were positively correlated with SLEDAI score in both lupus male subjects (*r* = 0.774 and *P* = 0.014) and female subjects (*r* = 0.812 and *P* = 0.049) (Figure 1C).

### EndoCAb IgM Levels and Proportions Are Reduced in SLE Patients

**Figure 2:**
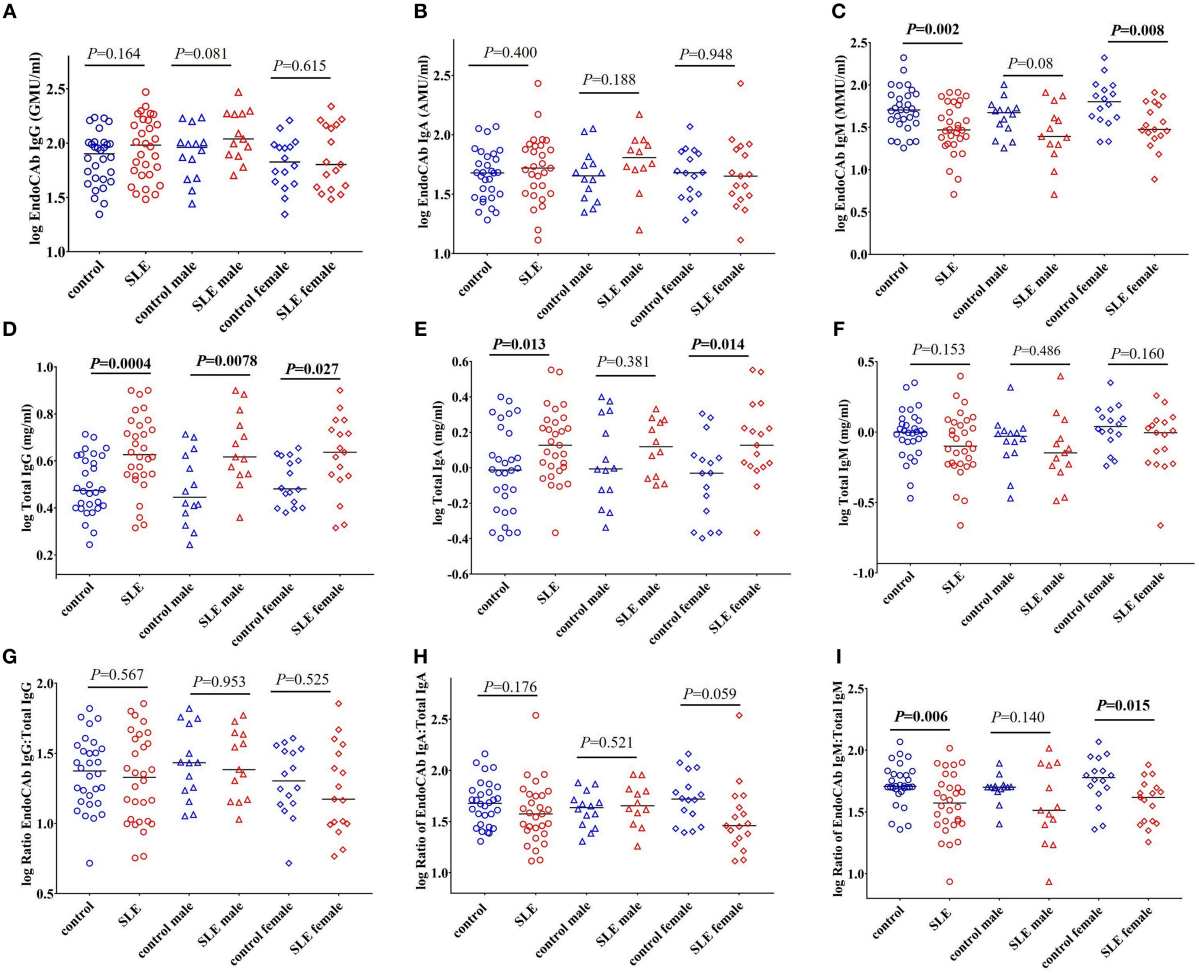
EndoCAb IgM levels are reduced in SLE subjects. **(A)** Levels of circulating EndoCAb IgG in healthy controls and SLE patients. **(B)** Circulating EndoCAb IgA levels in healthy controls and SLE subjects. **(C)** Levels of circulating EndoCAb IgM in healthy controls and SLE patients. **(D)** Levels of circulating total IgG in healthy controls and SLE patients. **(E)** Levels of circulating total IgA in healthy controls and SLE patients. **(F)** Levels of circulating total IgM in healthy controls and SLE patients. **(G)** Ratio of EndoCAb IgG:total IgG in healthy controls and SLE patients. **(H)** Ratio of EndoCAb IgA:total IgA in healthy controls and SLE patients. **(I)** Ratio of EndoCAb IgM:total IgM is reduced among SLE patients compared to healthy controls. For all figures, bars represent median analyte levels.

EndoCAbs are antibodies directed against the endotoxin core of LPS (28). They bind and neutralize circulating LPS. Alterations in the levels of EndoCAbs occur upon exposure to LPS and serve as a measure of bacterial exposure (20, 28, 29). Evaluation of EndoCAbs revealed that there is no significant differences in the levels of EndoCAb IgG and EndoCAb IgA between control and SLE patients (Figures 2A,B). Interestingly, we observed a significant decrease in EndoCAb IgM levels in SLE subjects (*P* = 0.002) compared to controls (Figure 2C). Gender comparisons revealed that SLE females had reduced levels of EndoCAb IgM (*P* = 0.008), whereas SLE males only showed a trend toward significance (*P* = 0.08). We next measured the concentration of total immunoglobulins (Igs) in the SLE and control cohorts. The results revealed a significant increase in the levels of total IgG (*P* = 0.0004) and total IgA (*P* = 0.013) in SLE patients compared to healthy controls (Figures 2D,E), confirming earlier observations (30, 31). SLE females showed an elevation of total IgA compared to control females (*P* = 0.014), whereas SLE males didn't show a significant change compared to control males (*P* = 0.381). Both SLE males (*P* = 0.0078) and SLE females (*P* = 0.027) showed a significant elevation in the levels of total IgG compared to their respective controls. We did not find a significant difference in total IgM levels (*P* = 0.153 healthy controls vs. SLE patients; *P* = 0.486 control males vs. SLE males; *P* = 0.160 control females vs. SLE females) (Figure 2F).

In order to determine whether the proportion of EndoCAbs were altered in SLE subjects; we determined the ratios of EndoCAb IgA:total IgA, EndoCAb IgG:total IgG, and EndoCAb IgM:total IgM between the groups. We did not find a significant change in the ratios of EndoCAb IgG:total IgG (*P* = 0.567 healthy controls vs. SLE; *P* = 0.953 control male vs. SLE male; *P* = 0.525 control female vs. SLE female). However, EndoCAb IgA:total IgA levels showed a trend toward reduction in females but not in males (*P* = 0.176 healthy controls vs. SLE; *P* = 0.521 control male vs. SLE male; *P* = 0.059 control female vs. SLE female) between the groups (Figures 2G,H). Interestingly, we observed a significantly reduced ratio of EndoCAb IgM:total IgM in SLE subjects compared to healthy controls (*P* = 0.006) (Figure 2I). SLE females showed reduced EndoCAb IgM:total IgM ratios (*P* = 0.015), whereas in males the difference was not significant (*P* = 0.140).

**Figure 3:**
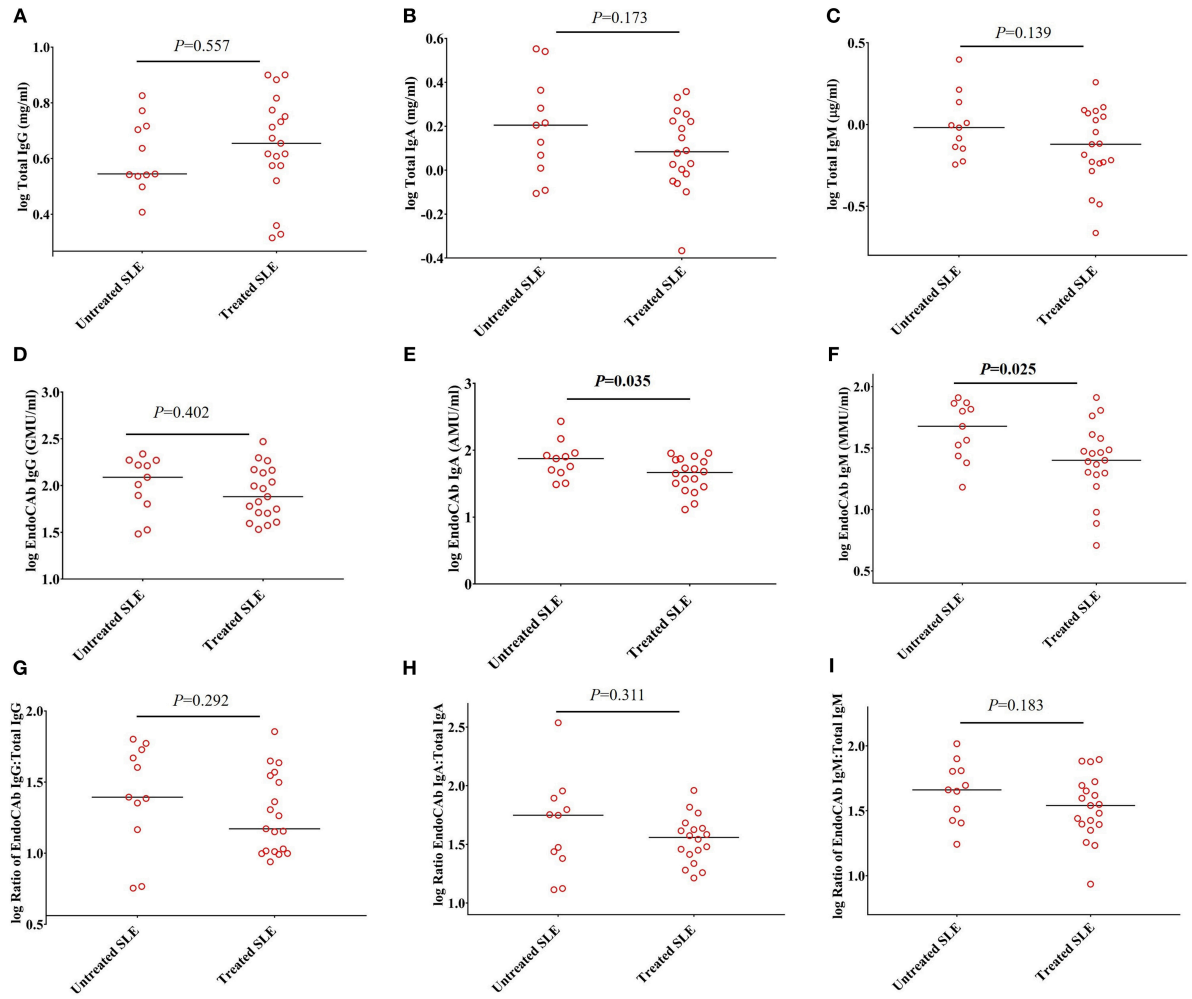
Ratios of EndoCAbs:total immunoglobulins are not affected by disease modulating drugs. **(A–C)** Concentrations of total immunoglobulins in SLE subjects (*n* = 19) treated with immunosuppressive and/or immunomodulatory drugs such as hydroxychloroquine, mycophenolate mofetil and/or azathioprine (*n* = 19) compared with untreated SLE subjects (*n* = 11). **(D–F)** Concentrations of EndoCAbs in untreated SLE patients (*n* = 11) and those treated with immunosuppressive drugs (*n* = 19). **(G–I)** Ratios of EndoCAbs:total Igs in SLE patients in untreated SLE subjects (*n* = 11) and those treated with immunosuppressive and/or immunomodulatory drugs (*n* = 19). Bars represent median analyte levels for all figures.

To determine whether these observations could be explained by the effects of immunosuppressive and/or immunomodulatory drugs, we compared the levels of total Igs, EndoCAbs, and the ratios of EndoCAbs:total Igs between treated and untreated SLE subjects. Total IgG (P = 0.557), IgA (P = 0.173), IgM (P = 0.139), and EndoCAb IgG (P = 0.402) levels were the same in SLE patients untreated and treated with immunosuppressive and/or immunomodulatory drugs (Figures 3A–D). However, EndoCAb IgA and EndoCAb IgM levels were found to be reduced significantly in SLE patients treated with immunosuppressive drugs compared to untreated SLE patients (*P* = 0.035 & *P* = 0.025, respectively) (Figures 3E,F). Importantly, the ratio of the three EndoCAbs:total Igs was not affected by immunosuppressive drugs in SLE subjects (Figures 3G–I).

**Figure 4:**
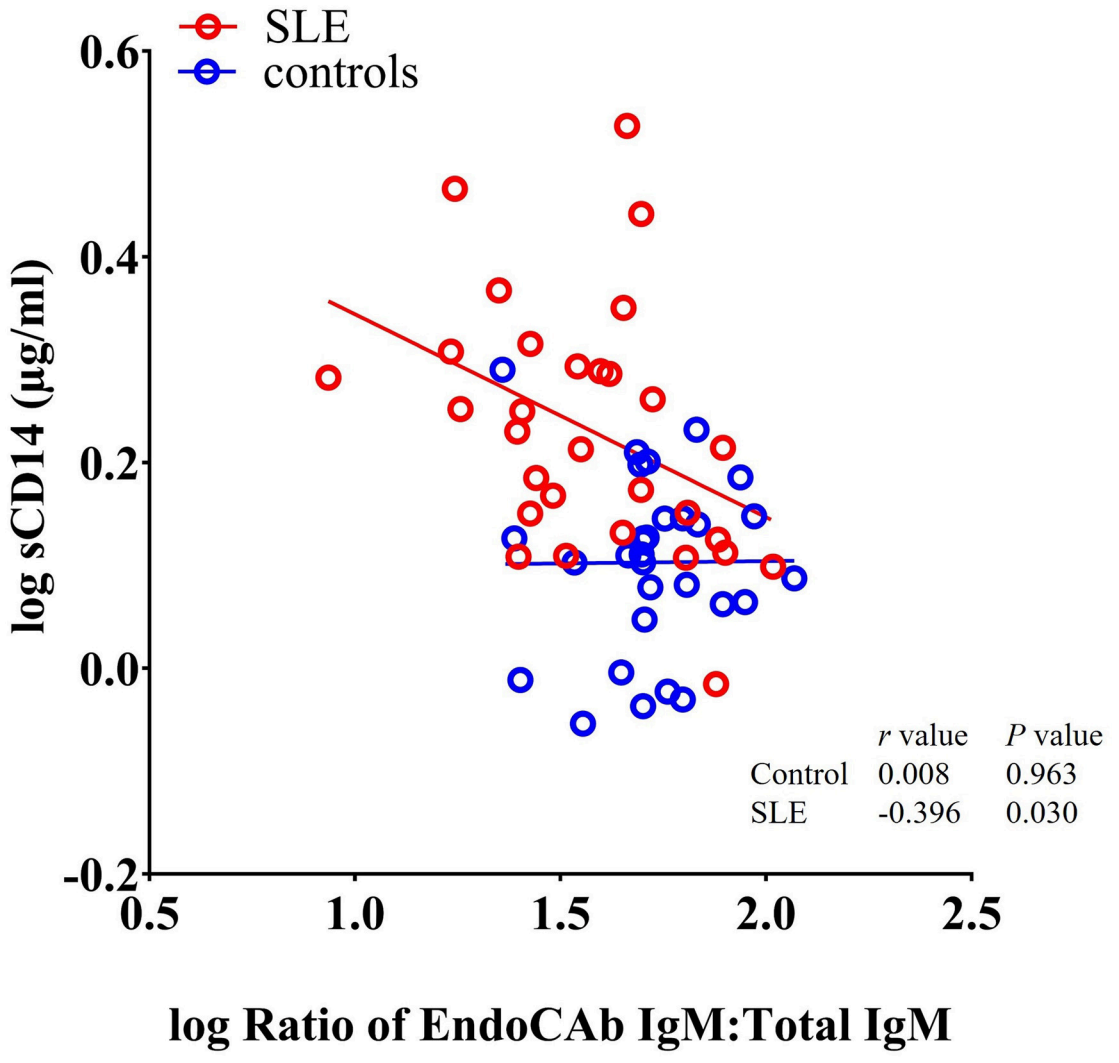
sCD14 levels correlate with the ratio of EndoCAb IgM:total IgM in SLE patients. Analysis showing a significant negative correlation between sCD14 and the ratio of EndoCAb IgM:Total IgM in SLE patients.

Since, both EndoCAbs and sCD14 are responsive to LPS, we asked whether the ratios of EndoCAbs:total Igs were associated with levels of sCD14 in SLE. We found a significant negative correlation between the ratio of EndoCAb IgM:total IgM and sCD14 (*P* = 0.030; *r* = −0.396) (Figure 4). Since bacterial exposure reduces EndoCAb IgM and elevates sCD14, this relationship is not surprising.

### Lysozyme Levels Are Increased in SLE Patients

**Figure 5:**
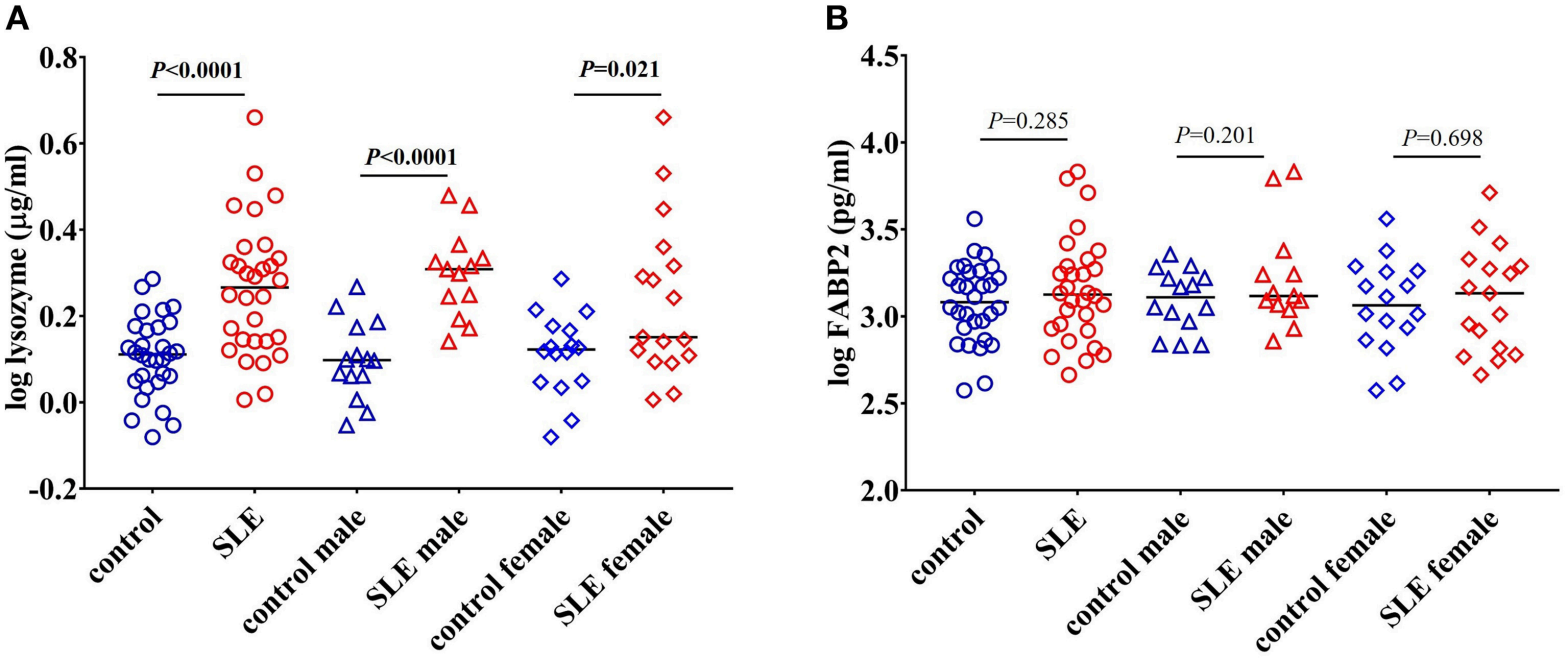
Elevated levels of lysozyme in SLE subjects. **(A)** Levels of circulating lysozyme in healthy controls and SLE patients. **(B)** FABP2 levels remain unchanged in healthy controls and SLE patients. Bars represent median analyte levels for all figures.

Peptidoglycan comprises a high proportion of the cell walls of Gram-positive and Gram-negative bacteria (32). Lysozyme is an important antimicrobial enzyme that hydrolyses the glycocidic bond of the peptidoglycan to kill microorganisms (33). Lysozyme is produced by monocytes, macrophages, neutrophils, and glandular cells (33). Levels of lysozyme are elevated in many diseases characterized by gut hyperpermeability including celiac disease, colitis, and Crohn's disease, suggesting that microbial flora upregulates this enzyme (33, 34). We tested lysozyme levels and found a significant increase in SLE patients (*P* < 0.0001) compared to controls. Both SLE males (*P* < 0.0001) and females (*P* = 0.021) exhibited a significant elevation when compared to controls (Figure 5A).

### Concentrations of FABP2 Are Not Different in SLE Patients

Changes in the levels of FABP2 in the circulation reflects epithelial cell loss and alterations in enterocyte turnover rate in the intestine. Thus, FABP2 serves as a useful marker in assessing intestinal permeability (20). We found that the levels of FABP2 were not different in these SLE subjects (*P* = 0.285 healthy controls vs. SLE patients; *P* = 0.201 control males vs. SLE males; and *P* = 0.698 control females vs. SLE females) (Figure 5B).

### Lysozyme Levels Positively Correlate With sCD14, LBP, and FABP2 Levels

**Figure 6:**
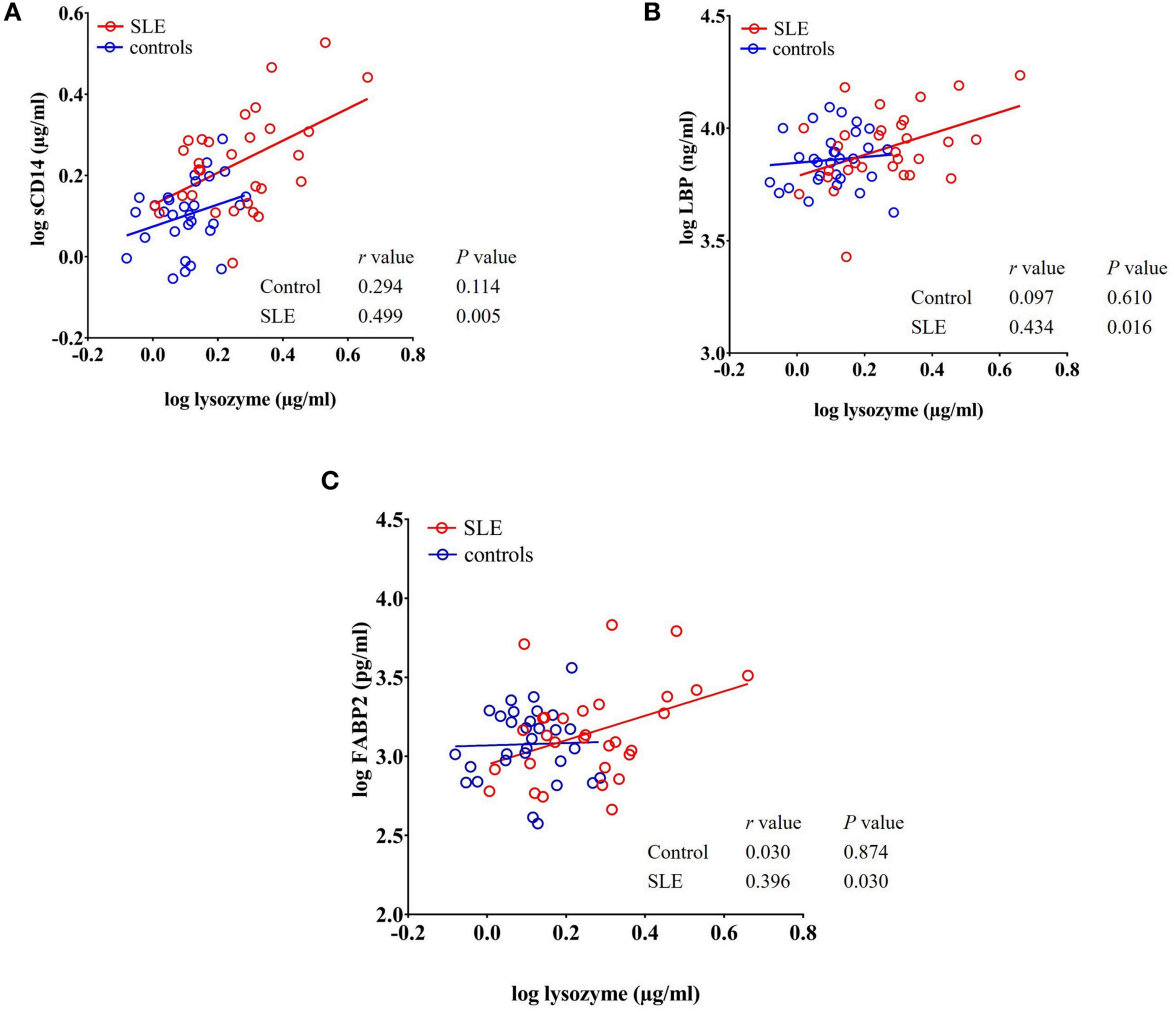
Circulating levels of lysozyme correlate with sCD14, LBP, and FABP2 in SLE patients. **(A–C)** Analysis showing a significant positive correlation of lysozyme with sCD14, LBP, and FABP2 in SLE patients.

We observed a significant positive correlation between the antibacterial protein lysozyme with sCD14 (*r* = 0.499, *P* = 0.005), LBP (*r* = 0.434, *P* = 0.016), and FABP2 (*r* = 0.396, *P* = 0.030) in SLE patients as determined by Pearson's r analysis. We did not observe any correlation between these factors in healthy controls (Figures 6A–C). Even though there is no statistical difference in LBP and FABP2 levels in SLE, their correlation with lysozyme suggests a common stimulus.

### Galectin-3 Levels in Healthy Controls and SLE Cohorts

**Figure 7:**
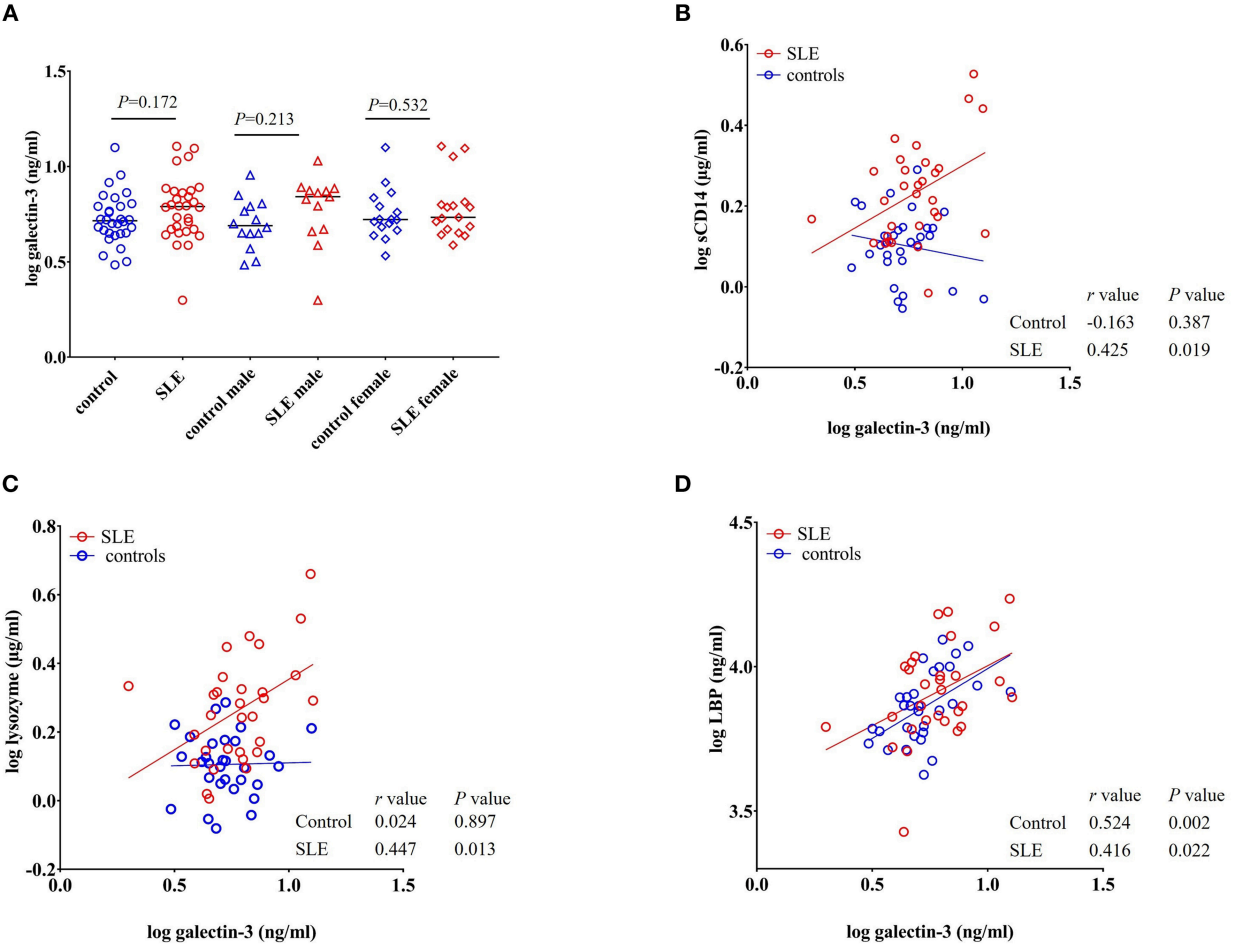
Galectin-3 levels correlate with sCD14, lysozyme and LBP in SLE subjects. **(A)** Plasma galectin-3 levels were not significantly different in SLE patients compared to healthy controls. Bars represent median analyte levels. **(B–D)** Correlation of galectin-3 levels with sCD14, lysozyme, and LBP in healthy controls and SLE patients. A significant positive correlation with sCD14, lysozyme, and LBP was observed in SLE patients. In healthy controls, a positive correlation was observed with galectin-3 vs. LBP **(D)** and a non-significant negative correlation was observed with galectin-3 vs. sCD14 **(B)**.

Galectin-3 is a multifunctional β-galactoside-binding protein produced by macrophages, monocytes, and dendritic cells (35). Previous reports demonstrated its upregulation in autoimmune disorders such as rheumatoid arthritis, Behcet's disease, and SLE (36–38). Exposure to bacterial products is suggested to induce the secretion of galectin-3 and the interaction between galectin-3 and LPS potentiates inflammation (39–41). Moreover, galectin-3 is involved in the regulation of immune and inflammatory responses (42). In our study, we found that galectin-3 levels were not statistically significant (*P* = 0.172 healthy controls vs. total SLE; male SLE subjects (*P* = 0.213); female SLE subjects (*P* = 0.532) (Figure 7A).

### Galectin-3 Levels Positively Correlate With sCD14 and Lysozyme in SLE Subjects

A significant positive correlation was seen for galectin-3 levels with sCD14 (*r* = 0.425, *P* = 0.019), lysozyme (*r* = 0.447, *P* = 0.013), and LBP in SLE patients (*r* = 0.416, *P* = 0.022). We did not observe such a positive correlation in healthy controls for lysozyme and sCD14. However, galectin-3 vs. LBP (*r* = 0.524, *P* = 0.002) was positively correlated in normal subjects. Interestingly, a negative correlation was observed between galectin-3 and sCD14 in healthy controls, but the correlation did not achieve statistical significance (*r* = −0.163, *P* = 0.387) (Figures 7B–D).

### Concentrations of LL-37 in SLE Cohort Were Not Different Than Those in Healthy Controls

**Figure 8:**
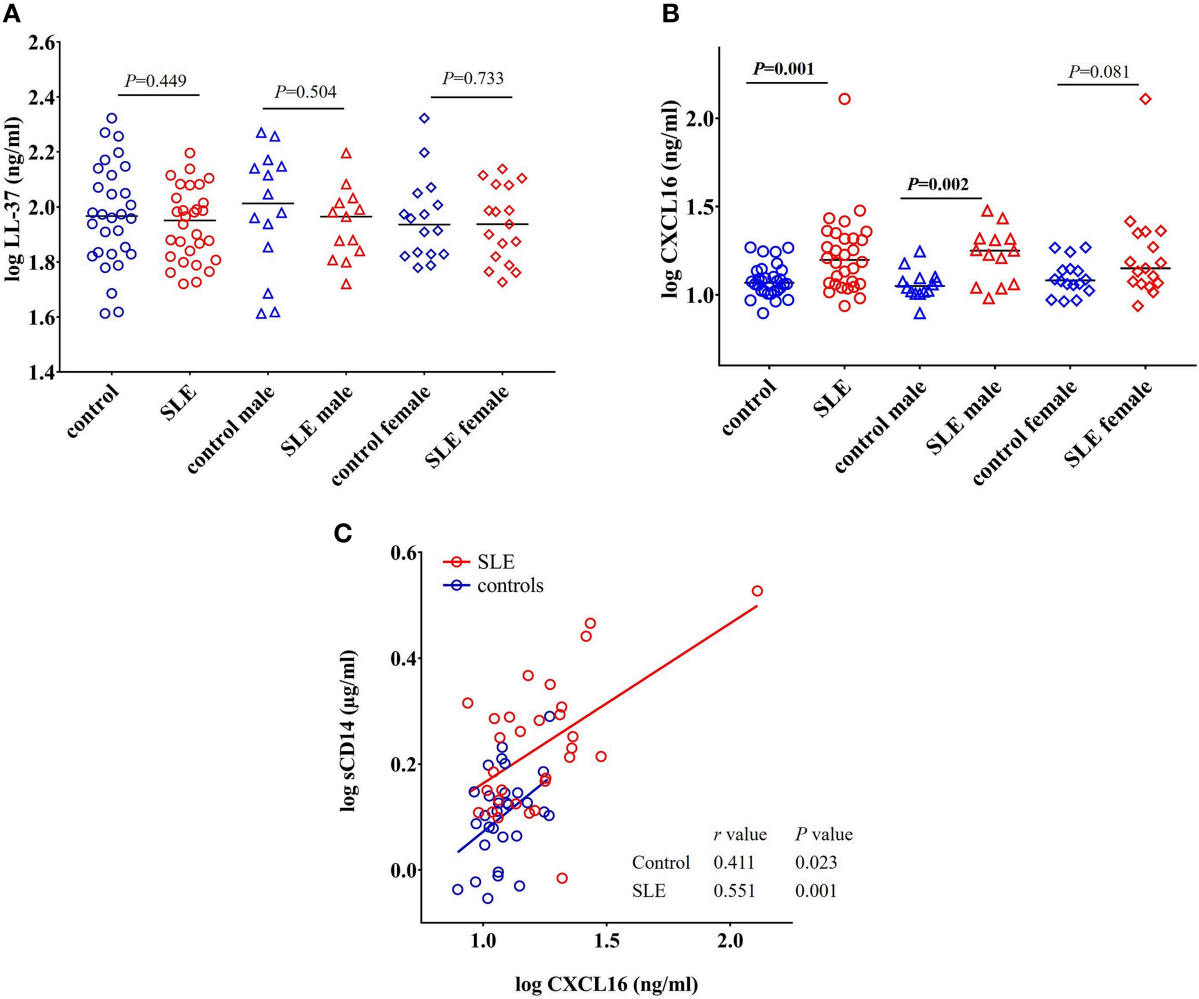
Concentrations of CXCL16 are elevated in SLE subjects. **(A)** Concentration of LL-37 remains unchanged in SLE patients compared with healthy controls and SLE patients. **(B)** SLE patients have elevated levels of CXCL16 levels compared to healthy controls. **(C)** CXCL16 levels were positively correlated with sCD14 levels in both SLE patients and healthy controls.

LL-37 is an antimicrobial peptide produced in response to bacteria and their products (43, 44). LL-37 can act as either a pro- or anti-inflammatory peptide depending on the context in which it is involved (45). We did not find any significant change in the concentration of LL-37 in SLE patients when compared to healthy controls (*P* = 0.449), SLE males compared to control males (*P* = 0.504), and SLE females compared to control females (*P* = 0.733) (Figure 8A).

### Levels of CXCL16 Are Increased in SLE Patients and Positively Correlated With sCD14 Levels

CXCL16 is an important multifunctional antimicrobial protein secreted by macrophages and dendritic cells and is involved in the phagocytosis of bacteria (46, 47). We observed a significant increase in the levels of CXCL16 (*p* = 0.001) in SLE patients when compared to control subjects (Figure 8B). SLE males were significantly different from control males (*P* = 0.002) whereas SLE females only showed a trend toward significance (*P* = 0.081).

A significant positive correlation was observed between CXCL16 levels and sCD14 levels in both SLE patients (*r* = 0.551, *P* = 0.001) and healthy controls (*r* = 0.411, *P* = 0.023) (Figure 8C). This could be due to the involvement of a common stimulus driving the elevation of both CXCL16 and sCD14 levels.

## Discussion

In this investigation, we demonstrated coordinated changes in ARFs in SLE patients. Constituents from Gram-negative and Gram-positive bacteria including LPS, peptidoglycan, flagellin, endogenous lipids, and lipoteichoic acid (LTA) can stimulate monocyte/macrophage activation and induce ARFs (48, 49). ARFs are predominantly expressed and produced by monocytes/macrophages, neutrophils, hepatocytes, and B cells (23, 49–53) which initiate a cascade of protective immunoregulatory and proinflammatory factors potentiating bacterial clearance. This mechanism could initiate or accelerate SLE.

We found that sCD14 levels were increased in SLE, confirming previous studies (23, 54, 55). Recent reports demonstrated that elevated levels of sCD14 in plasma are associated with intestinal barrier dysfunction (56, 57). The positive correlation between sCD14 and disease activity (SELENA-SLEDAI score) levels suggests a role for endotoxemia in SLE progression. LPS exposure could thus account for many of the findings in this study. sCD14 levels are increased in other bacterial infection-associated diseases including sepsis, arthritis, periodontitis, and kidney diseases. Elevated levels of sCD14 in the circulation indicates a systemic response to bacterial exposure (58–61). Correlation of sCD14 with SELENA-SLEDAI in SLE indicates that sCD14 could be an acute phase protein. Furthermore, it is known that excess sCD14 levels reduce the LPS-monocyte interaction and thereby decrease the detrimental effects of systemic leukocyte activation (60, 62). Increased levels of sCD14 in the circulation promote the release of LPS from cells. Due to the rapid binding of LPS with monocytes/macrophages, released LPS can further stimulate the production of proinflammatory cytokines. LPS is also transferred to lipoproteins for its neutralization (60). Interestingly, endogenous lipoproteins cannot neutralize LPS quickly due to their slow binding rate (63). The biological effects of sCD14 appear to be concentration dependent and further investigations are required to understand the effects of sCD14 mediated release of cell bound LPS.

We did not observe any significant changes in the levels of LBP, a marker in the LPS-CD14 pathway, in SLE cohort compared to healthy controls. The absence of a significant change in LPS-binding molecules could be due to differences in affinity, production, or clearance, or perhaps by the low molar ratio of LPS to these relatively abundant ligands (64). At the same time, increased levels of sCD14 levels could also be due to monocyte/macrophage activation by elevated levels of inflammatory cytokines (49). Since the elevated levels of sCD14 do not reflect the sole involvement of bacterial products, further studies are warranted to define the exact basis of elevated levels of sCD14 in lupus.

The reduction in the ratio of EndoCAb IgM:IgM levels is particularly provocative, since this immunoglobulin directly neutralizes LPS. To our knowledge, this is the first report analyzing EndoCAb levels in SLE. EndoCAb IgM antibody levels are reduced during an antimicrobial response and act as a sensitive marker of recent endotoxin exposure (20, 28, 64). It was hypothesized that binding and neutralization of endotoxin by EndoCAb IgM leads to its subsequent degradation, reflecting its reduced levels (65). Thus, EndoCAb IgM antibody titres are inversely proportional to acute endotoxemia (66, 67). We observed reduced levels of EndoCAb IgM and elevated levels of sCD14 in the SLE cohort. Moreover, a negative correlation between sCD14 and the ratio of EndoCAb IgM:Total IgM also links the EndoCAb mediated clearance of LPS. Since elevated levels of sCD14 in plasma enhance LPS release from monocytes, it is quite likely that the reduction of EndoCAb IgM is due to the binding of free endotoxins. Alternatively, sustained depletion of EndoCAb IgG is found during chronic endotoxemia including sepsis (28). Currently, it is unknown about the initiating events and the exact fate of endotoxin-EndoCAb complex and/or whether this complex has any specific role in the activation of the complement pathway. Follow-up studies are pertinent to address this question.

The significant increase in the levels of total IgA and IgG observed in this SLE cohort could be due to immune activation. Previous studies also reported elevated levels of IgA and IgG in lupus patients (30, 31). It was shown that higher amounts of immunoglobulin secreting cells in the peripheral blood are responsible for the elevated levels of IgA and IgG in SLE (68). Moreover, IgG plays an important role in the formation of immune complexes in SLE, which induce a chronic inflammatory condition (69, 70). Elevated levels of IgA could result as an immune response from the mucosal immune system against translocated bacterial products (71). Even though the ratios of EndoCAb IgA:total IgA and EndoCAb IgG:total IgG were not significantly different among the groups, the ratio of EndoCAb IgA:total IgA in SLE females showed a trend toward significant reduction suggesting exposure of bacterial products from the gut.

Immunosuppression may lead to increased bacterial growth and infection (72, 73). Increased exposure to microbial products could be due to gut leakiness and/or infection that could result from immunosuppression. It was previously reported that immunosuppression can somewhat reduce the levels of EndoCAbs but the results were not statistically significant (74). In this report, we showed that EndoCAb IgM levels were lower in SLE subjects treated with immunosuppressive and/or immunomodulatory drugs that included hydroxychloroquin, mycophenolate mofetil, and azathioprine. However, the ratio of EndoCAb IgM:Total IgM was significantly reduced in SLE subjects. This suggests a specific effect on EndoCAb IgM indicating increased LPS exposure. The negative correlation between sCD14 and the ratio of EndoCAb IgM:total IgM suggests bacterial exposure and monocyte/macrophage activation.

We found elevated levels of lysozyme in SLE patients. Previous studies reported an elevation of this enzyme in response to microbial exposure and alterations in intestinal permeability (33, 34, 75). Bacterial exposure increases monocyte/macrophage and neutrophil activation (76, 77). Activated macrophage produce proinflammatory cytokines such as IL-6 and TNF-α can increase the production of lysozyme (33, 78). Although we did not find significant differences in LBP or FABP2 levels, we observed significant positive correlations between lysozyme levels and sCD14, LBP and FABP2. This suggests a relationship and possible common stimulus for these factors. Interestingly, the positive correlations between these factors appear to be novel and have never been reported in SLE.

Our analyses also indicated that the SLE cohort possesses increased levels of CXCL16 compared to healthy controls. CXCL16 is an important chemokine participating in host defense and acts as a mediator of innate immune responses (79). Recent studies showed the involvement of CXCL16 in SLE patients (51, 80). In addition to macrophages and dendritic cells, CXCL16 is also produced by keratinocytes, where it plays an antimicrobial role (79). Qin et al. (51) reported higher levels of CXCL16 in SLE patients with cutaneous disease compared to subjects without cutaneous involvement. We did not observe any correlation between CXCL16 levels and disease activity in male and female SLE patients (data not shown). Endotoxemia also increases the levels of CXCL16 (81). Increased levels of CXCL16 in SLE cohort might be due to the exposure of bacterial products. CXCL16 is recognized as a novel class of scavenger receptor. The role of scavenger receptors in the uptake of modified LDL is well-reported (82). Moreover, involvement of CXCL16 in the phagocytosis of both Gram-negative and Gram-positive bacteria was also documented (46). A positive correlation between CXCL16 and sCD14 in our study further substantiate the involvement of bacterial products for the elevation of CXCL16 in SLE patients. Binding of LPS with CD14 leads to the activation of various signaling cascades including NF-κB and drives the induction of CXCL16 (81). It is reported that LPS binds to lipoproteins and the resulting LPS-lipoprotein complex undergoes receptor-mediated endocytosis (63, 83). As a scavenger receptor, elevated levels of CXCL16 appears to be a compensatory mechanism to neutralize LPS-lipoprotein complexes. Considering these observations, it is likely that the increased level of CXCL16 in SLE cohorts results from bacterial exposure. Follow-up studies will be necessary to clarify the functional role of CXCL16 in the transport of bacterial products and its receptor-mediated neutralization.

Galectin-3 acts as a regulator of innate immunity or as a pattern-recognition receptor (84, 85). Plasma galectin-3 levels have been reported to be significantly elevated in SLE (38). However, our analysis of galectin-3 levels did not reveal significant alterations. The differences in results between these studies could reflect the male to female ratio or disease severity. Shi et al. (38) used a predominantly female cohort with a SLEDAI score average of 12.5 as compared to our cohort, which included 43% males, and subjects with an average SLEDAI score of 4.9. Yet following gender stratification of our SLE subjects, the female cohort showed no signs of increase in galectin-3 levels. Moreover, we did not observe a correlation between disease activity and galectin-3 levels (data not shown). Therefore, further studies are required to elucidate the role galectin-3 in the pathology of SLE. Interestingly, a positive correlation between galectin-3 with sCD14, lysozyme, and LBP in our study suggests the involvement of bacterial exposure as a common stimulus.

SLE primarily affects women. There has been a lot of progress in delineating the pathophysiology of the disease, but the female predominance is still unexplained (1). Interestingly, we observed some gender differences in ARF levels. For example, EndoCAb IgM levels were reduced in females, whereas males only showed a trend. Conversely, CXCL16 levels were increased in male SLE subjects, whereas females showed a trend. Males and females show different levels of innate immunity and the alterations in innate immunity lead to the production of auto-antigens and autoimmunity in SLE (86–88). Various studies indicated the involvement of immune responses from intestinal mucosa in determining gender differences in SLE (89). Differences in gut microbiome between males and females could be a possible reason for gender-specific changes in the levels of ARFs in this study. Gender-specific differences in gut microbiome composition have been widely reported (90–92). Furthermore, microbiota composition differs between males and females in other autoimmune conditions, which further demonstrate a possible connection between gender, the gut microbiome and immunity (90, 93–95). It is suggested that sex-specific changes in the composition of gut microbiota may be a contributing factor for more severe lupus symptoms in females (90).

We believe that the unifying stimulus for the observed differences in ARF levels in SLE subjects is bacterial exposure. Several factors, such as increased intestinal permeability, alterations in gut flora, and changes in the morphology of the intestine could contribute to the heightened antimicrobial response observed in our study. In human SLE, a relative increase in the abundance of Lachnospiraceae and a lower ratio of Firmicutes to Bacteroidetes was demonstrated (14–16, 96). Alterations in microbiota may inhibit intestinal barrier functions, triggering inflammation and antimicrobial defense mechanisms (97). It was shown that the receptors for LPS and peptidoglycans (TLR4 and TLR2, respectively) are elevated in SLE, which promote pathology and autoantibody production (13, 98, 99).

Although we observed no significant change in the levels of FABP2 (marker of compromised intestinal integrity), we did find a positive correlation between FABP2 and lysozyme, suggesting the involvement of bacterial exposure in SLE cohort. Due to broad variance in the levels of FABP2, the difference may be hard to detect with this sample size. In the absence of a significant change in FABP2 levels, measuring circulating LPS, zonulin, and intestinal biopsy are alternatives for assessing compromised gut integrity. However, none of these approaches is without pitfalls. For example, circulating LPS and zonulin levels are not considered reliable markers for compromised gut permeability due to technical difficulties in quantification (57, 100–103). The acquisition of intestinal tissue from SLE patients and controls is uncommon, which makes such an approach less feasible. For these and other reasons, assessment of biomarkers related to microbial exposure and immune activation is considered one of the best possible measures of analyzing compromised gut permeability in humans using archived serum or plasma samples (57, 104).

**Figure 9:**
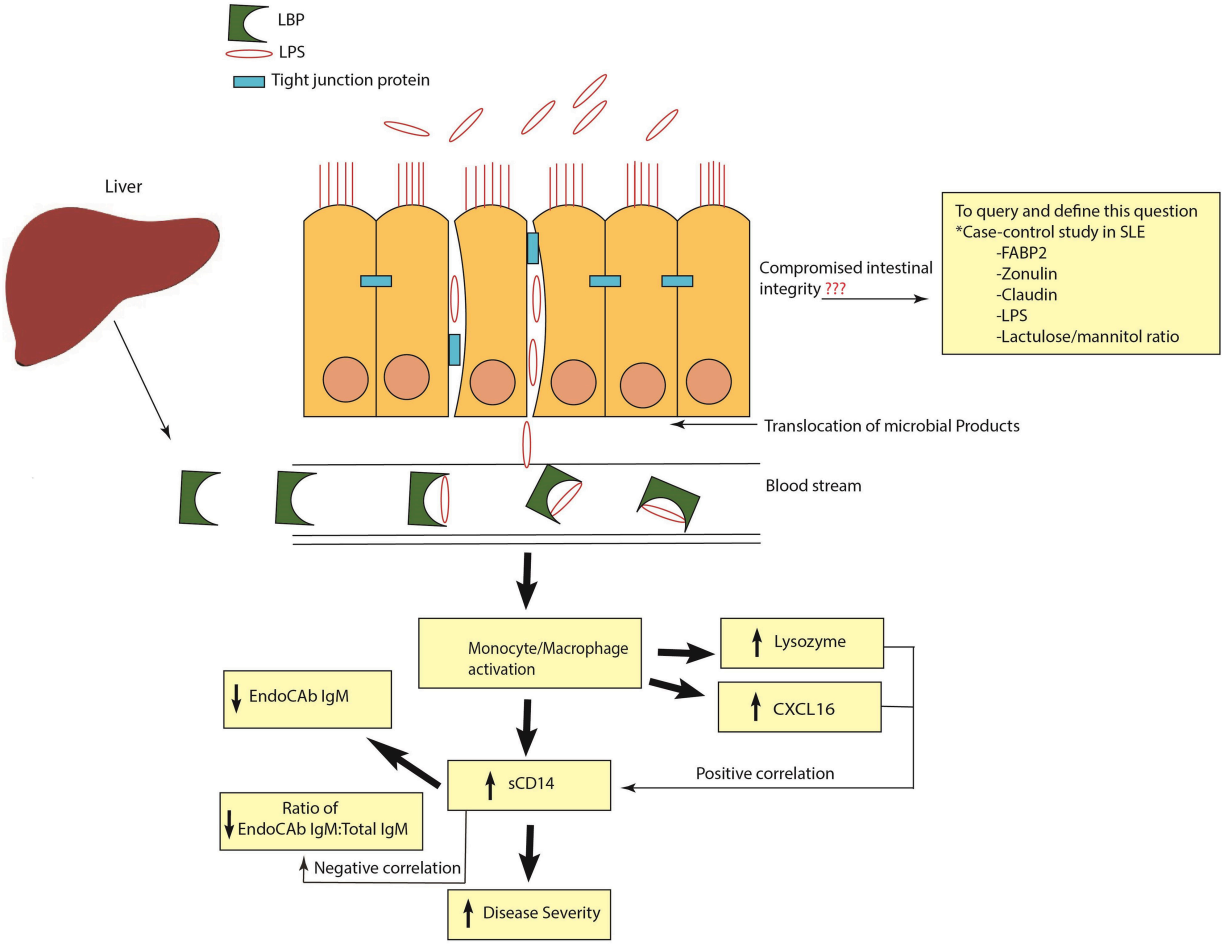
Schematic representation of heightened antimicrobial response in SLE. Release of LPS and other microbial products into systemic circulation causes monocyte/macrophage activation and the release of antimicrobial response factors (ARFs). Elevated levels of sCD14 are an indication of monocyte/macrophage activation. High sCD14 can also accelerate the release of cell bound LPS from monocytes. Binding and neutralization of free endotoxins by EndoCAb IgM causes its reduction in the circulation. In addition, other ARFs such as lysozyme and CXCL16 are induced to combat and neutralize the bacterial products in the circulation. A positive correlation of sCD14 with lysozyme and CXCL16 and a negative correlation between sCD14 and the ratio of EndoCAb IgM:Total IgM suggests that exposure to microbial products is a common stimulus for the induction of these ARFs. Compromised intestinal integrity is the likely reason for the translocation of microbial products into the circulation. In order to query and define this, case-control studies in SLE analyzing the suite of different markers of intestinal permeability are essential.

In conclusion, our results demonstrate a heightened antimicrobial response with marked elevations and correlations of ARFs in SLE (Figure 9). Based on our results, it appears that inhibiting microbial exposure may dampen monocyte/macrophage activation and improve therapeutic outcomes for SLE patients. Indeed, pre-clinical studies showed that antibiotic treatment ameliorated systemic autoimmunity, increased intestinal epithelial barrier function and eliminated pathogenic autoantibodies and T cells in SLE (7, 8). However, a better understanding of the linkage between SLE and heightened antimicrobial responses could provide novel therapeutic approaches for the management of this disease in high risk populations.

## Author Contributions

PA: experimental design and execution, data analysis, and manuscript preparation; RH and NS: experimental design, data analysis, and reviewed the manuscript; JB: recruited subjects and reviewed the manuscript.

### Conflict of Interest Statement

The authors declare that the research was conducted in the absence of any commercial or financial relationships that could be construed as a potential conflict of interest.
